# Unveiling the Nitrogen-Doping Mechanism in Carbon Catalysts for Oxidative Dehydrogenation of Ethanol to Acetaldehyde

**DOI:** 10.3390/ma18102345

**Published:** 2025-05-18

**Authors:** Lingxin Kong, Chenxi Guo, Wenkai Song, Yujie Liu, Guiyao Luo, Yan Xu, Yujun Zhao, Peng Jin

**Affiliations:** 1 Key Laboratory for Green Chemical Technology of Ministry of Education, Collaborative Innovation Center of Chemical Science and Engineering, School of Chemical Engineering and Technology, Tianjin University, Tianjin 300072, China; konglingxin@tju.edu.cn (L.K.); wenkai_song@tju.edu.cn (W.S.); liuyujie@tju.edu.cn (Y.L.); 3022207260@tju.edu.cn (G.L.); xuyan040506@tju.edu.cn (Y.X.); 2 School of Materials Science and Engineering, Hebei University of Technology, Tianjin 300130, China; 202321801056@stu.hebut.edu.cn

**Keywords:** acetaldehyde, carbon catalysts, ethanol, nitrogen-doped, oxidative dehydrogenation

## Abstract

Oxidative dehydrogenation (ODH) of ethanol to acetaldehyde is an important production process. However, it still suffers from low deactivation, selectivity, and high costs. Herein, we developed a new strategy for preparing mesoporous nitrogen-doped carbon catalysts by carbonization of phenolic resin with silica as a hard template. The catalyst demonstrated an impressive acetaldehyde selectivity of over 76% at 270 °C for 25 h during the ODH of ethanol to acetaldehyde. Mechanistic studies have shown that the two carbon atoms in adjacent C=O groups are replaced by nitrogen atoms in the N_0_-Gra-O structural unit. The C=O functional group on the surface of the catalyst is the active center for the ODH of ethanol to acetaldehyde, and the introduction of nitrogen atoms can reduce the adsorption capacity of acetaldehyde molecules at the active site (Δ*G* values can be reduced by 0.11–0.45 eV), enabling rapid desorption of the product and avoiding the problem of excessive oxidation, thereby improving the selectivity of acetaldehyde. This work reveals the structure–activity relationship between active sites and selective regulation of nitrogen-doped carbon-based catalysts for the ODH of ethanol, providing a theoretical basis for the development of efficient non-metallic carbon-based catalysts.

## 1. Introduction

Acetaldehyde is in high demand within the chemical industry [1,2], and approximately 85% of commercial acetaldehyde is obtained by the Wacker–Hoechst process [3,4]. The Wacker–Hoechst process primarily relies on precious metal catalysts (PdCl_2_-Cu-Cl_2_), uses ethylene as the reactant, and is conducted in a tank reactor under highly acidic conditions. In contrast, the ethanol dehydrogenation process offers significant advantages in terms of catalyst cost, environmental sustainability, and capital expenditure for equipment. Furthermore, the use of bioethanol as a reactant can significantly decrease the reliance on petroleum resources in the acetaldehyde production process, which aligns with the principles of green and sustainable development. In recent years, advancements in coal-based ethanol and bioethanol technologies have facilitated the emergence of ethanol-based acetaldehyde production as a significant and environment-friendly alternative to the Wacker–Hoechst process. Therefore, oxidative/non-oxidative dehydrogenation of ethanol to acetaldehyde is receiving increasing attention.

As is well known, carbon catalysts, including carbon nanotubes [5], carbon nanofibers [6,7], and graphene [8], have been extensively utilized in a wide range of heterogeneous catalytic reactions. These catalysts have demonstrated exceptional performance in the oxidation of various chemicals, such as ethylbenzene [9], alcohols [7,10], and alkanes [11,12]. They possess several distinct advantages, including tunable surface properties with respect to acidity, basicity, and electron density [10]. Regarding the ODH of ethanol to acetaldehyde, the C=O functional groups were the active sites [13] when carbon nanotubes were used as catalysts in this reaction. Furthermore, both pyridinic nitrogen and graphitic nitrogen exert significant influence on the adsorption of ethanol and the dissociative adsorption of O_2_ [5]. Additionally, optimizing the ratio between surface pyridinic nitrogen and graphitic nitrogen on the catalyst surface is of critical importance, and this ratio should be in the range of 0.7–1.0 [14]. Notably, oxidized multi-walled carbon nanotubes exhibited a higher apparent activation energy (E_a_) for CO_x_ formation compared to formaldehyde formation during methanol conversion, leading to enhanced formaldehyde selectivity [15]. Based on these findings, carbon catalysts may exhibit a higher selectivity for the ODH of ethanol to acetaldehyde; however, the carbon nanotubes and other materials described above show good catalytic performance for the ODH of ethanol. However, several challenges include scalability issues during preparation [16], high preparation costs, significant pressure drop in fixed-bed reactors [17], and complex shaping processes, which collectively pose substantial barriers to the industrial development of carbon catalysts. Meanwhile, the mechanisms by which nitrogen-doped carbon catalysts influence activity and selectivity remain to be further elucidated at the atomic level.

Herein, we report a method of nitrogen-doped mesoporous carbon catalysts for the oxidative dehydrogenation (ODH) of ethanol to acetaldehyde. The catalyst was synthesized through the carbonization of a phenolic resin modified with nitrogen-containing compounds, using silica sol as a hard template. Notably, this catalyst exhibited an outstanding acetaldehyde selectivity of over 76% at 270 °C for 25 h. Energy Dispersive Spectrometry (EDS) and X-ray photoelectron spectroscopy (XPS) confirm the successful doping of nitrogen. The presence of nitrogen atoms on the catalyst surface can influence the microenvironment of the active sites in chemical reactions. By correlating the number of C=O on the catalyst surface with the conversion rates of ethanol, it was found that there is a good linear relationship. These results directly unveil that the C=O functional groups on the catalyst surface serve as active sites for this reaction. Acetaldehyde temperature-programmed desorption (acetaldehyde-TPD-MS) and density functional theory (DFT) calculations demonstrate that nitrogen doping reduces the adsorption capacity of acetaldehyde molecules and enhances the selectivity of acetaldehyde. This work reveals the structure–activity relationship between active sites and selective regulation of nitrogen-doped carbon-based catalysts for the ODH of ethanol, providing a theoretical basis for the development of efficient non-metallic carbon-based catalysts.

## 2. Experiment Section

The chemicals, catalyst preparation method, catalyst characterization, calculation methods, catalyst activity test conditions, and product analysis are detailed in the Supporting Information.

## 3. Results and Discussion

### 3.1. Synthesis and Characterization of N-Doped Carbon Catalysts

**Scheme 1:**
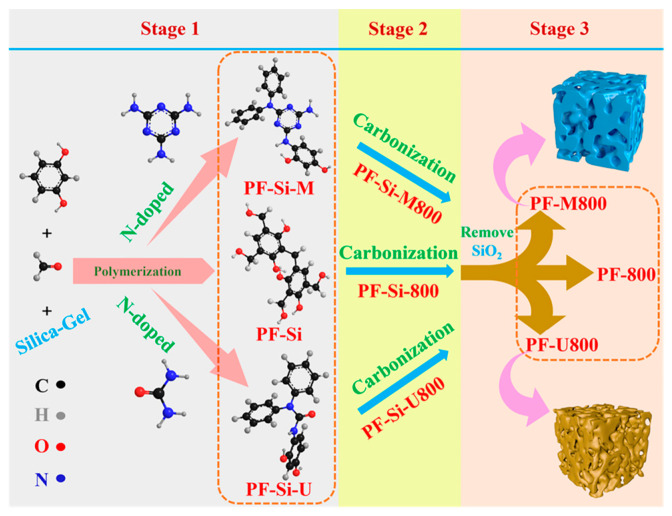
Preparation of N-doped carbon catalysts.

Scheme 1 shows the preparation process of the current N-doped carbon catalysts. Under alkaline conditions, resorcinol reacts with formaldehyde to form a polyhydroxyphenol mixture during the heating process at 40 °C. Regarding the mechanism of nitrogen doping, a hydroxymethylation reaction occurs when formaldehyde reacts with active hydrogen in the amino group [18,19]. Then, nitrogen-containing compounds with hydroxyl groups can undergo dehydration reactions with polyhydroxyphenols, thereby introducing nitrogen into the carbon precursors. After the carbonization and removal of SiO_2_, N-doped mesoporous carbon was successfully obtained.

**Figure 1:**
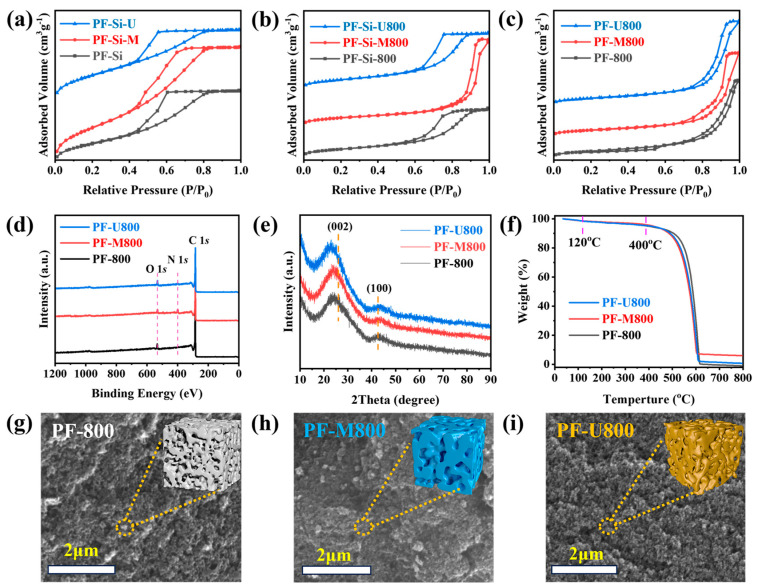
(**a**–**c**) Three stages of the catalyst synthesis process for these samples: N_2_ adsorption−desorption isotherm; (**d**–**f**) XPS spectra, XRD patterns, and TG analysis for PF-800, PF-M800, and PF-U800, respectively; (**g**–**i**) SEM images for PF-800, PF-M800, and PF-U800.

The catalyst synthesis process was divided into three stages. In the first stage, polymerization occurred, and the samples were named PF-Si-M (utilizing melamine as a nitrogen source), PF-Si (without nitrogen doping), and PF-Si-U (using urea as a nitrogen source). FT-IR analyses were performed on these samples (Figure S1 in the Supplementary Materials). The infrared absorption peak at 800 cm^−1^ is assigned to 1,4-disubstituted benzene, while the peak at 1112 cm^−1^ is attributed to -OH in primary alcohol. Additionally, the absorption peak at 1220 cm^−1^ is ascribed to C-O in phenolic hydroxyl groups [20]. Significant differences are observed among the infrared absorption spectra of PF-Si, PF-Si-M, and PF-Si-U samples. The infrared absorption peak at 1547 cm^−1^ is attributed to the formation of C=N bonds resulting from melamine polymerization in PF-Si-M. The infrared absorption peak at 1620 cm^−1^ is ascribed to the C=O bond, which can be attributed to the polymerization of urea and formaldehyde. In contrast to the PF-Si sample, both PF-Si-M and PF-Si-U exhibit distinct zigzag amino peaks (3100–3750 cm^−1^) [21], thereby confirming the successful doping of nitrogen. The N_2_ adsorption–desorption isotherms of these samples and their pore size distribution curve are depicted in Figure 1a and Figure S2a. Table S1 (in the Supplementary Materials) summarizes the BET surface areas, pore volumes, and average pore diameters. The N_2_ adsorption–desorption isotherms of these samples show IV-type isotherms [22] with H2-type hysteresis loops, indicating a mesoporous catalyst structure. The triangular hysteresis loops, characterized by a steep desorption branch, suggest an inkpot-like pore structure. As the relative pressure increases, the adsorption capacity in the capillary condensation region also rises, further supporting this morphological interpretation.

The second stage involved carbonization, resulting in the formation of PF-Si-M800 (utilizing melamine as a nitrogen source), PF-Si-800 (without nitrogen doping), and PF-Si-U800 (using urea as a nitrogen source). As shown in Figure 1b, PF-Si-800 and PF-Si-U800 retain the inkpot-shaped structure, whereas the inkpot-shaped morphology of PF-Si-M is lost during the carbonization process owing to the partial decomposition of melamine. This conclusion can be further verified through the BET surface areas (Table S1) and the pore size distribution curve (Figure S2b in the Supplementary Materials).

Finally, in the third stage, the hard template SiO_2_ was removed to obtain the catalyst samples PF-M800 (utilizing melamine as a nitrogen source), PF-800 (without nitrogen doping), and PF-U800 (using urea as a nitrogen source). All these samples exhibit type-IV isotherms with H1-type hysteresis loops, indicating a mesoporous structure for these carbon catalysts (Figure 1c). The specific surface areas approach approximately 1000 m^2^/g, while the pore volumes reach around 3.5 cm^3^/g. The range of pore sizes distributed falls within 10–15 nm (Table S1). The XPS spectra in Figure 1d show characteristic nitrogen peaks for both PF-M800 and PF-U800. This reinforces the evidence of successful nitrogen doping. The XRD patterns in Figure 1e show that the peaks observed at angles of 26° and 43° correspond to the (002) and (100) lattice planes of typical nanocarbon materials [23]. The PF-800, PF-M800, and PF-U800 have amorphous morphology (Figure 1g–i), and the surfaces of the nitrogen-containing catalysts are evenly distributed (Figure S3 in the Supplementary Materials). The nitrogen contents of PF-M800 and PF-U800 are significantly higher than that of PF-800 (0 at% for nitrogen contents), as evidenced by EDX elemental maps with values of 3.1 and 1.1 at%, respectively. The EDX elemental maps are consistent with the FT-IR spectrum (Figure S1) and XPS spectrum (Figure 1d). The oxidation resistance of the carbon catalysts was further characterized by TG analysis. As illustrated in Figure 1f, the weight loss below 120 °C can be attributed to the removal of adsorbed water. The catalysts exhibit negligible weight loss below 400 °C under the flow of 10% O_2_/N_2_, indicating their exceptional antioxidant performances.

### 3.2. Structure–Activity Relationship

**Figure 2:**
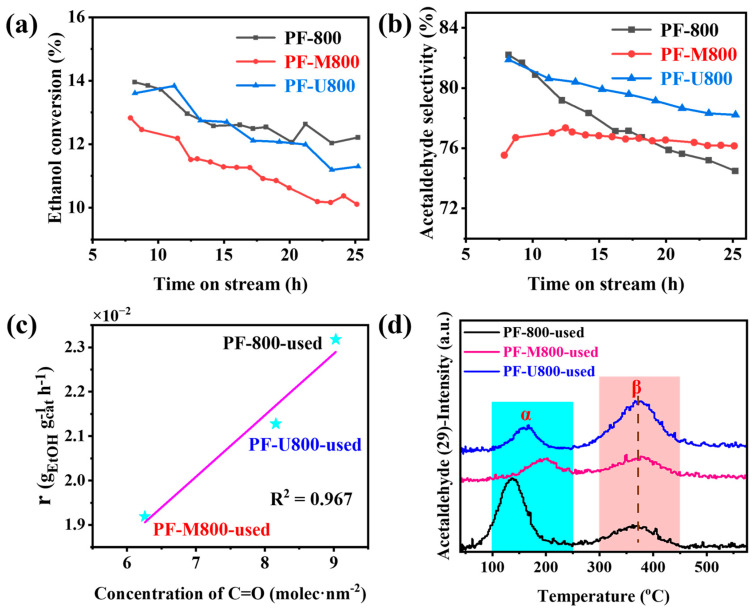
Catalytic performance of PF-800, PF-M800, and PF-U800. (**a**) Ethanol conversion and (**b**) acetaldehyde selectivity. Reaction conditions: catalyst = 0.5 g, 270 °C, WHSV = 0.19 h^−1^, ethanol = 1 vol%, oxygen = 2 vol%, total flow: 78 mL·min^−1^ (helium as the balance); (**c**) correlation between ethanol conversion and concentration of C=O groups on the used catalysts; (**d**) acetaldehyde-TPD-MS profiles for the used catalysts (PF-800, PF-M800 and PF-U800).

Nitrogen-doped mesoporous carbon catalysts were used to evaluate the catalytic performance of the ODH of ethanol. Figure 2a,b illustrate the catalytic performance of ethanol. The ethanol conversions for PF-800, PF-M800, and PF-U800 are 12.2%, 10.1%, and 11.3%, respectively. The calculated deactivation rate constants are determined by the following equation:

(1) $$K_{d}\left(h^{-1}\right)=\frac{\ln((1-X_{\mathrm{final}})/X_{\mathrm{final}})\;-\;\ln((1-X_{\mathrm{initial}})/X_{\mathrm{initial}})}{h}$$

where K_d_ represents the deactivation rate constant; X represents the conversion of ethanol for PF-800, PF-M800, and PF-U800, which are 0.009, 0.016, and 0.013, respectively.

Generally, the deactivation rate constant of the catalyst is relatively low, indicating that the catalyst exhibits good stability. The acetaldehyde selectivity for these three catalysts is 74.5%, 76.2%, and 78.2%, respectively. The acetaldehyde selectivity of the nitrogen-doped catalysts exhibits higher and more stable acetaldehyde selectivity than PF-800 after 18 h of reaction. The stability of the PF-M800 catalyst in terms of acetaldehyde selectivity is significantly higher than that of the other two catalysts. This enhanced stability may be attributed to the unique structure of melamine, which demonstrates greater stability compared to urea [24]. The nitrogen atoms on the surface of the PF-M800 catalyst may effectively stabilize the catalyst surface or inhibit the generation of acidic groups on the catalyst surface. The byproducts of PF-800, PF-M800, and PF-U800 are ethene, ethyl ether, ethyl formate, and ethyl acetate, respectively (Figure S4 in the Supplementary Materials). These catalysts exhibit higher selectivity for ethyl acetate compared to other byproducts. However, different nitrogen sources have different effects on the performance of the catalyst. Therefore, the catalyst surface was subjected to XPS analysis to gain a deeper understanding of the changes in the content, valence states, and species distribution of various elements in both fresh and used catalyst samples. Figure S5a (in the Supplementary Materials) shows the deconvolution of the O 1s spectra into four distinct high-resolution peaks: quinone, C=O, C-O, and C-OH [25]. Combined with Tables S2 and S3 (in the Supplementary Materials), the oxygen species content on the catalyst surface increases after the reaction, probably owing to the conversion of defect sites to other oxygenated groups. As illustrated in Figure 2c, there is a clear linear correlation between the ethanol conversion rate and the concentration of carbonyl groups (C=O) on the used catalysts. This suggests that C=O is the main active site of the catalyst.

Nitrogen species have a significant effect on the acid-basicity and electron clouds, which affect the product selectivity of alcohol oxidation [26]. Therefore, the crucial role of nitrogen species cannot be underestimated. The N 1*s* spectra in Figure S5b exhibit distinct peaks corresponding to various nitrogen species, including N_1_ (pyridinic N), N_2_ (pyrrolic N), N_3_ (graphitic N), and N_4_ (oxidized N) [25,26]. Combined with the results in Table S3, it is obvious that although the nitrogen-doped catalyst shows higher acetaldehyde selectivity, there is no obvious linear correlation between the content of nitrogen species and acetaldehyde selectivity. Therefore, acetaldehyde selectivity may be related to the type of nitrogen species. To further characterize the adsorption–desorption properties of acetaldehyde on the catalyst surface, acetaldehyde-TPD-MS analysis was performed. As shown in Figure 2d and Table S4 (in the Supplementary Materials), catalyst surfaces exhibit two distinct types of adsorption sites for acetaldehyde: α at the range of 100–250 °C for weak adsorption sites and β at the range of 300–450 °C for strong adsorption sites. After integrating peaks α and β, their corresponding adsorption capacities can be obtained. As listed in Table S4, the adsorption capacities of the weak adsorption site at the peak α for PF-M800-used and PF-U800-used are only 0.015 mmol/g and 0.012 mmol/g, respectively, which are significantly lower than that of PF-800-used (0.042 mmol/g). These results indicate that the introduction of nitrogen can significantly reduce the number of weak sites for acetaldehyde adsorption. The presence of nitrogen species facilitates the desorption of acetaldehyde from the catalyst surface and effectively inhibits side reactions, resulting in an increased acetaldehyde selectivity. The difference in acetaldehyde selectivity may be primarily caused by this factor. It is worth noting that PF-U800 has the least amount of acetaldehyde adsorption at the peak α, which might be attributed to its unique type of nitrogen species structure on the catalyst surface. The amounts of acetaldehyde desorbed from the β-sites are determined to be 0.067 mmol/g for PF-800-used, 0.046 mmol/g for PF-M800-used, and 0.065 mmol/g for PF-U800-used. Interestingly, nitrogen-doped catalyst samples such as PF-U800-used exhibit similar behavior as the undoped samples (PF-800-used) in terms of the desorption amount of acetaldehyde at β-sites. This suggests that the impact of strong adsorption sites (β-sites) on acetaldehyde selectivity can be neglected.

To gain a deeper understanding of the experimental observations and elucidate the mechanism of action of nitrogen species, further DFT calculations were conducted. According to the XPS results, several porous graphene catalyst models with C=O active sites and different nitrogen doping structures were constructed and optimized. The optimized structures of different catalyst models are shown in Figure S6 (in the Supplementary Materials). The adsorption of an acetaldehyde molecule was then calculated for each of them. Gra-O represents the catalyst model with undoped nitrogen atoms. N_0_-Gra-O means that the two adjacent positions of C=O are replaced by nitrogen atoms, which should be easily formed when urea is used as the nitrogen source. The N_1_-Gra-O, N_2_-Gra-O, and N_3_-Gra-O correspond to the catalyst models where a carbon atom is replaced by pyridine nitrogen, pyrrole nitrogen, and graphite nitrogen near the C=O, respectively. Gra-O-C_2_H_4_O* represents the most stable adsorption structure of the acetaldehyde molecule on Gra-O, and Δ*G* is the corresponding Gibbs free energy change during adsorption. The “-C_2_H_4_O*” in the name of Gra-O-C_2_H_4_O* represents the state of acetaldehyde adsorption near the C=O site. Other adsorption structures of catalyst models are similarly named. 

**Figure 3:**
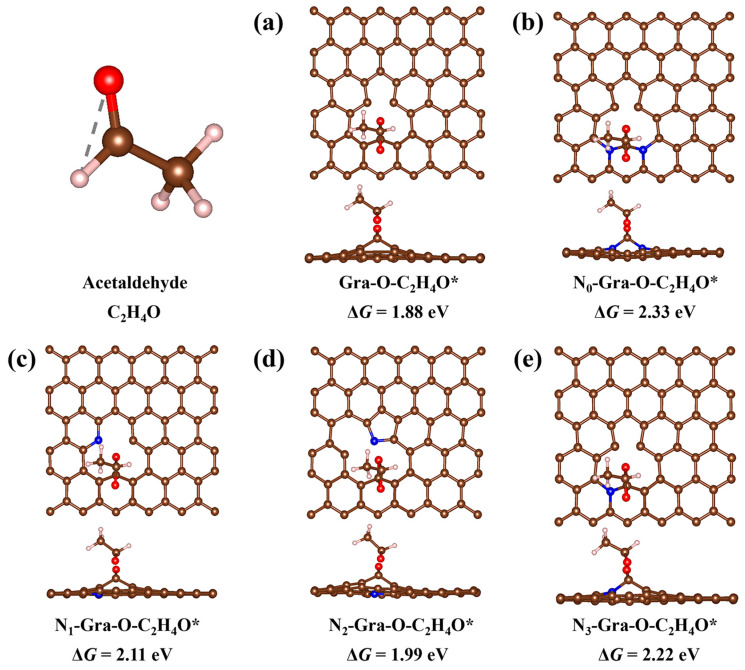
(**a**–**e**) optimized structures of different catalysts with an adsorbed acetaldehyde molecule (top and side views) and corresponding Δ*G* values upon adsorption. C: brown; O: red; N: blue; H: white.

During the adsorption calculations, various molecular orientations were systematically considered. As shown in Figure 3, the calculated Δ*G* values follow the order of Gra-O (1.88 eV) < N_2_-Gra-O (1.99 eV) < N_1_-Gra-O (2.11 eV) < N_3_-Gra-O (2.22 eV) < N_0_-Gra-O (2.33 eV). Thus, the N doping can greatly facilitate the desorption of the final acetaldehyde product, where N_0_-Gra-O with two N dopants is the most favorable one for the desorption. The desorption capacities of acetaldehyde by N_1_ (pyridinic N) and N_3_ (graphitic N) exhibit remarkable similarity. Therefore, the doping of carbon with urea presents a higher acetaldehyde selectivity since it should boost the formation of the N_0_-Gra-O structure during the carbonization process.

### 3.3. Chemical Properties of Nitrogen Doping Catalyst

In addition to acetaldehyde adsorption and desorption on the catalyst surface, factors such as ethanol adsorption capacity, oxygen activation, surface defects, and acid–base properties of carbon materials may influence catalytic performance [5,14,25,27]. Therefore, we conducted a series of experiments and characterization analyses on various catalysts.

**Figure 4:**
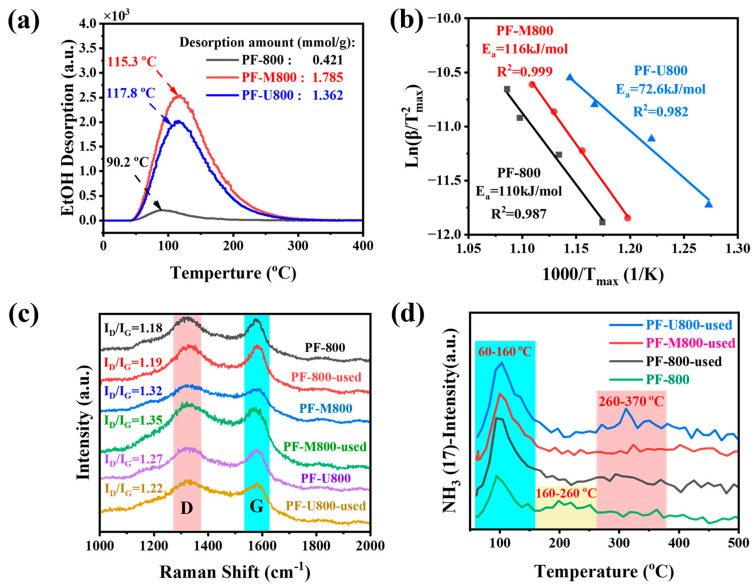
Characterization of various carbon catalysts: (**a**) EtOH-TPD-MS with the upper right corner showing desorption amount of ethanol; (**b**)activation energy for oxygen; (**c**) Raman spectra changed before and after the reaction; (**d**) NH_3_-TPD-MS for various carbon catalysts.

EtOH-TPD-MS was conducted on these catalysts using ethanol as the adsorbent agent. The positions of the desorption peaks represent the interaction intensity between the ethanol and the catalyst surface, while the peak area signifies the amount of ethanol adsorbed. As depicted in Figure 4a, the desorption peak positions for PF-800, PF-M800, and PF-U800 are 90.2 °C, 117.8 °C, and 115.3 °C, respectively. The desorption amounts of PF-M800 and PF-U800 are 1.785 mmol/g and 1.362 mmol/g, respectively, significantly exceeding that of PF-800 (0.421 mmol/g). These results demonstrate that the introduction of nitrogen species significantly enhances the interactions between the catalyst surface and ethanol, as well as the adsorption capacity of ethanol [25].

Nitrogen doping, particularly at the edge sites, can also significantly enhance the adsorption and dissociation of O_2_, thereby facilitating the formation of active oxygen centers. The nitrogen doping also has an electron-donating effect, which makes it easier to activate O_2_ [28]. The oxygen adsorbed on N-doped carbon nanotubes showed an interesting electron configuration similar to that of the active oxygen anion [29]. Therefore, differential thermogravimetry (DTG) was employed to characterize the above carbon catalysts at different heating rates, aiming to investigate the activation energy (E_a_) of oxygen adsorption on them. Figure S7 and Table S5 (in the Supplementary Materials) show the DTG curves of these carbon catalysts at different heating ramps. The DTG peak value (T_m_, K) and heating rate (β, K·min^−1^) were utilized in accordance with the Kissinger–Akihiro–Sunose equation [5]:

ln(β/T^2^_m_) = ln(AR/E_a_) − E_a_/RT_m_ (2)

According to Figure 4b, PF-U800 exhibits the lowest E_a_ value of 72.6 kJ·mol^−1^. This may be because of its unique precursor urea structure, which makes nitrogen atoms more likely to appear near C=O, and thus significantly enhances oxygen activation on the catalyst surface [25]. In contrast, PF-M800 exhibits a much higher E_a_ value of 116 kJ·mol^−1^. The distinctive phenyl-like ring structure of melamine facilitates the preferential localization of doped nitrogen atoms at non-active sites, thereby hindering the adsorption and activation of O_2_. As a result, this leads to an increased stability of nitrogen atoms on the carbon surface. Moreover, it is noteworthy that there is no correlation between E_a_ values for O_2_ activation and catalytic activity. Combined with Figure 4a, it can be concluded that the adsorption of ethanol and the activation of O_2_ may not be the rate-determining step for the ODH of ethanol.

As for the influence of surface defects, Raman spectroscopy is the primary method for evaluating surface graphitization in carbon materials, while peak fitting analysis provides detailed insights into the defect structures. The Raman spectra in Figure 4c show the changes before and after the reaction. The Raman profile ranging from 1100 to 1750 cm^−1^ can be categorized into five bands, namely D2, D, D1, G, and D’ (the details can be found in Table S6 (in the Supplementary Materials) [5,25]. Both PF-M800 and PF-U800 exhibit higher I_D_/I_G_ values (1.32 and 1.27, respectively) than PF-800 (1.18), indicating a significant increase of surface defects on the nitrogen-doped catalysts. By comparing the Raman spectral data before and after the reaction, no significant change in surface graphitization is observed for the surface defects on these catalysts. Additionally, the I_D_/I_D’_ value can also be used to estimate the type and content of carbon defects [30]. As shown in Figure S8a–c (in the Supplementary Materials), the I_D_/I_D’_ values of the fresh catalysts (PF-800 and PF-U800) are 29.21 and 18.3, respectively. According to the defect theory [30], the primary defective species for these two samples is mainly sp^3^ hybridization [25]. PF-M800 has the lowest I_D_/I_D’_ value of 8.9, indicating that the vacancy-like defects, instead of sp^3^ hybridization, are the primary defective species [25,30]. For the used samples of PF-800 and PF-U800, the number of sp^3^-defects exhibit a significant decrease in comparison with corresponding fresh samples, while the I_D_/I_D’_ values of PF-M800 slightly increase after the reaction. It suggests that the vacancy-like defects are more stable during the reaction than the sp^3^ hybridization defects. This could be one key reason for the superior stability of acetaldehyde selectivity achieved on PF-M800 (in conjunction with Figure 2b).

NH_3_-TPD-MS characterization was conducted to further elucidate the changes in surface acidity. Figure 4d shows the presence of two distinct types of acidic sites on the catalyst surface, namely weak and middle-strong acidic sites. When the results of the fresh and used PF-800 catalysts are compared, a notable augmentation in the quantity of weak acidic sites can be found on the used catalyst within the temperature range of 60 to 160 °C, whereas there is an enhancement in middle-acidic sites between 160 and 260 °C. These results indicate an increase in both the strength and quantity of acidic sites during the reaction. Moreover, it is worth noting that there are significant discrepancies in ammonia desorption among different used catalysts within the temperature range of 260–370 °C. PF-M800 exhibits a lower peak intensity than PF-800 and PF-U800, indicating that its surface nitrogen species inhibit the conversion of partial functional groups into acidic groups during the reaction process. To further elucidate the types and quantities of oxygen-containing functional groups on the catalyst surface, the Boehm titration was employed for characterizing the catalyst. As listed in Table S7 (in the Supplementary Materials) and taking fresh PF-800 as the reference, after the oxidation reaction, the quantities of -COOH and phenolic hydroxyl groups increase significantly from 0.265 mmol/g and 0.164 mmol/g to 0.442 mmol/g and 0.767 mmol/g, respectively. These results further confirm the formation of more acid groups on the catalyst surface during the oxidation reaction. It is widely acknowledged that increased acidity facilitates the dehydration of ethanol to produce byproducts [31]. However, even though the number of acid functional groups on PF-800-used is lower than that on PF-U800-used, PF-800-used does not present a higher acetaldehyde selectivity than PF-U800-used. Therefore, the special nitrogen species (N_0_-Gra-O) on PF-U800-used influence the product of alcohol oxidation [26]. Consequently, it can be concluded that the significant difference in acetaldehyde selectivity is primarily attributed to the presence of nitrogen species rather than the acidity of the catalyst surface.

Additionally, the oxidation resistance of carbon catalysts after the reaction was further characterized through TG-MS analysis. As illustrated in Figures S8d–f, these carbon catalysts in the flow of 5% O_2_/N_2_ are presented, and a noticeable increase in sample mass is observed within the temperature ranges of 50–175 °C and 275–400 °C. This can be attributed to the oxidation reaction between oxygen and defect sites on the catalyst surface. The MS signal peak position of CO_2_ for the PF-M800 catalysts is much higher than that of PF-U800 (Figure S8e in the Supplementary Materials), indicating the relatively higher stability of PF-M800 in an oxygen environment. The presence of more sp^3^-defects lowers the ability of PF-U800 in oxidation resistance. Additionally, Figure S8f (in the Supplementary Materials) demonstrates the successful introduction of nitrogen into carbon material, which agrees well with the XPS and SEM results.

## 4. Conclusions

In this study, a series of N-doped mesoporous carbon catalysts were synthesized through the carbonization of N-modified phenolic resin using silica as a hard template. The catalyst exhibited an attractive high acetaldehyde selectivity of over 76% at 270 °C for 25 h. The activation energy analysis of O_2_ and ethanol-TPD-MS indicates that neither O_2_ activation nor ethanol adsorption serves as the rate-limiting step. Raman analysis reveals a structural transformation from sp^3^-defects to vacancy-like defects with minimal changes in graphitization during the reaction. The physicochemical properties of the catalysts were investigated by XPS, which revealed that the C=O was the active site. Based on the results of NH_3_-TPD-MS and the Boehm titration method coupled with catalytic performance analysis, it can be inferred that the acidity of the catalyst surface is unlikely to be the primary factor influencing the selectivity for acetaldehyde. Instead, nitrogen doping effectively reduces the adsorption capacity of carbon for acetaldehyde by acetaldehyde-TPD-MS, thereby achieving higher acetaldehyde selectivity. The nitrogen-doped carbon catalyst with urea presents a higher acetaldehyde selectivity because it can boost the formation of the N_0_-Gra-O structure during the carbonization process. DFT calculations further demonstrate that this structure has the strongest acetaldehyde desorption ability among various nitrogen species. It provides crucial theoretical support and guidance for the utilization of carbon-based catalysts in oxidative dehydrogenation, thus exhibiting significant potential for industrial implementation.

## Data Availability

The original contributions presented in this study are included in the article and Supplementary Materials. Further inquiries can be directed to the corresponding author.

## Supplementary Materials

The following supporting information can be downloaded at: https://www.mdpi.com/article/10.3390/ma18102345/s1, Figure S1: FT-IR spectra of PF-Si, PF-Si-M, and PF-Si-U.; Figure S2. Three stages of the catalyst synthesis process for these samples: Pore size distribution curve.; Figure S3. SEM images corresponding to EDX elemental maps of PF-800 (a), PF-M800 (b), and PF-U800 (c).; Figure S4. Selectivity of byproducts of PF-800 (a), PF-M800 (b), and PF-U800 (c).; Figure S5. XPS O 1*s* (a) and N 1*s* (b) deconvolution spectra for fresh and used PF-800, PF-M800, and PF-U800.; Figure S6 (a)–(e) optimized structures of different catalyst models (top and side views). C: brown; O: red; N: blue; H: white.; Figure S7. DTG curves at different heating ramps of PF-800 (a), PF-M800 (b), and PF-U800 (c).; Figure S8. The first-order Raman deconvolution spectra of PF-800 (a), PF-M800 (b), and PF-U800 (c) changed before and after the reaction; TG-MS profiles of the PF-800, PF-M800, and PF-U800 after the reaction (d) and the corresponding MS signals of CO_2_ (e), NO and NO_2_ (f).; Table S1: Physicochemical properties associated with three stages of catalyst synthesis process.; Table S2. XPS analysis of content of each element of carbon catalysts before and after the reaction.; Table S3. O 1s and N 1s XPS analysis results of carbon catalysts.; Table S4. Desorption amount and desorption temperature of acetaldehyde on various catalysts.; Table S5. DTG T_max_, ramp rate, and corresponding activation energy calculated according to Kissinger–Akahira–Sunose equation.; Table S6. Sample denotations and comparison of the fitting parameters from the peaks fitted in the Raman spectra of carbon-based catalysts before and after the reaction.; Table S7. The number of surface functional groups on carbon catalysts. References [32,33,34,35] are cited in the Supplementary Materials.
