# Supplementary material for: Unveiling the Nitrogen-Doping Mechanism in Carbon Catalysts for Oxidative Dehydrogenation of Ethanol to Acetaldehyde
Source: Materials (Basel). 2025 May 18;18(10):2345. doi: 10.3390/ma18102345 (PMC12112757; doi:10.3390/ma18102345)
Supplement: Supplementary file 1 [file materials-18-02345-s001.zip › materials-3622740-supplementary.pdf]

materials

MDPI

Supplementary Materials

Unveiling the Nitrogen-Doping Mechanism in Carbon Catalysts for Oxidative Dehydrogenation of Ethanol to Acetaldehyde

Lingxin Kong [1,†], Chenxi Guo [2,†], Wenkai Song [1], Yujie Liu [1], Guiyao Luo [1], Yan Xu [1], Yujun Zhao [1,*] and Peng Jin [2,*]

[1] Key Laboratory for Green Chemical Technology of Ministry of Education, Collaborative Innovation Center of Chemical Science and Engineering, School of Chemical Engineering and Technology, Tianjin University, Tianjin 300072, China
[2] School of Materials Science and Engineering, Hebei University of Technology, Tianjin 300130, China
* Correspondence: yujunzhao@tju.edu.cn (Y.Z.); china.peng.jin@gmail.com (P.J.)
† These authors contributed equally to this work.

1. Experiment section

1.1. Chemicals

Formaldehyde (HCHO, Shanghai Macklin Biochemical Co., Ltd., China, Shanghai.), resorcinol ($C_6H_6O_2$, AR, Shanghai Macklin Biochemical Co., Ltd., China, Shanghai.), silica sol (JN-30, Dezhou crystal fire technology glass Co., Ltd, China, Shandong.), sodium carbonate anhydrous (Na_2CO_3, AR, Tianjin Jiangtian Chemical Technology Co., Ltd., China, Tianjin.), sodium bicarbonate ($NaHCO_3$, AR, Tianjin Jiangtian Chemical Technology Co., Ltd., China, Tianjin.), urea ($CO(NH_2)_2$, AR, Tianjin Jiangtian Chemical Technology Co., Ltd., China, Tianjin.), melamine ($C_3H_6N_6$, AR, Aladdin), sodium hydroxide (NaOH, AR, Tianjin Jiangtian Chemical Technology Co., Ltd., China, Tianjin.), ethanol (CH_3CH_2OH, AR, Kemiou Chemical Reagent Co., Ltd., China, Tianjin.)

1.2. Catalyst Preparation

A total of 0.1215 g of Na_2CO_3 was dissolved in deionized water (4 mL) followed by the addition of 13.2678 g of formalin and 9 g of resorcinol. Then, the mixture was poured into a certain amount of silica sol and stirred at 40 °C for 2 h. The mixture was aged 72 h at 80 °C, and it was named PF-Si. After carbonization at 800 °C, PF-Si was transformed into sample PF-Si-800. Subsequently, this sample underwent treatment with NaOH solutions of specific concentrations at 70 °C for a duration of 6 h to eliminate SiO_2. Consequently, the resulting sample was designated as PF-800.

A total of 0.1215 g of Na_2CO_3 was dissolved in deionized water (4 mL) followed by the addition of 13.2678 g of formalin and 9g of resorcinol. This mixture was named A. Then, 10.038 g of melamine was dissolved in formalin (17.6904 g). This mixture was named B. Then, the mixture B was poured into mixture A and stirred at 40 °C for 2 h. The mixture was aged 72 h at 80 °C, and it was named PF-Si-M. After carbonization at 800 °C, the sample PF-Si-M800 was obtained. Then, this sample underwent treatment with NaOH solutions of specific concentrations at 70 °C for a duration of 6 h to eliminate SiO_2. Consequently, the resulting sample was designated as PF-M800.

A total of 0.1215 g of Na_2CO_3 was dissolved in deionized water (4 mL) followed by the addition of 13.2678 g of formalin and 9 g of resorcinol. This mixture was named A. Then, 3.2724 g of urea was dissolved in 17.6904 g of formalin, and this mixture was named B. Then, mixture B was poured into mixture A and stirred at 40 °C for 2 h. Then, the

 https://doi.org/10.3390/ma18102345

mixture was aged 72 h at 80 °C, and this sample was named PF-Si-U. After carbonization at 800 °C, the sample PF-Si-U800 was obtained. Then, this sample underwent treatment with NaOH solutions of specific concentrations at 70 °C for a duration of 6 h to eliminate SiO_2. Consequently, the resulting sample was designated as PF-U800.

1.3. Activity test and product analysis

The oxidative dehydrogenation experiment of ethanol was conducted under atmospheric pressure. A total of 0.5 g of catalyst was placed in the fixed-bed reactor. The reaction temperature was set to 270°C. The total gas flow rate was 78 mL/min, with ethanol accounting for 1 vol%, O_2 accounting for 2 vol%, and helium serving as the inert gas. The byproducts include ethylene, ethyl ether, ethyl formate, and ethyl acetate. An online gas chromatograph equipped with FID and TCD detectors (Anhui Chromatography GC5190, China) was used to monitor the products. The reaction tube is made of quartz. The blank experiment illustrated that the reaction of ethanol can be deemed insignificant.

1.4. Catalyst Characterization

The BET surface area, pore volume, and average pore diameter of the prepared catalysts were characterized on a Micromeritics TristarII 3000 analyzer at 77 K and calculated by using the Barrett–Joyner–Halenda (BJH) method. The contents of residual silica in the samples were analyzed by thermogravimetric analysis (TG, Netzsch TG209F3 apparatus) in air from 35 to 800 °C. X-ray diffraction (XRD) measurements were performed using a SmartLab (9kW) diffractometer, employing the graphite-filtered Cu Kα radiation (λ = 1.5406 Å) at room temperature. Scanning electron microscopy (SEM) images were obtained on a Hitachi S-4800 field emission scanning electron microscope. Fourier transform infrared (FTIR) spectroscopy was performed using a Nicolet 6700 spectrometer (ThermoFisher Company, America) with a scanning range from 400 to 4000 cm^{-1}. Raman experiments were conducted by Confocal Raman Microscopy (Horiba) with a laser wavelength of 532 nm. The activation energy of oxygen adsorption on various catalysts were performed on a Netzsch TG209F3 apparatus in a flow of 10% O_2/N_2 (100 mL/min) with different heating rates (5, 10, 15, 20 k/min) from 30 to 800 °C. The DTG peak value (T_m, K) and heating rate (β, K/min) were used according to the Kissinger–Akahira–Sunose equation: $\ln(\beta/T^2_m) = \ln(AR/E_a) - E_a/RT_m$.

Thermogravimetry (TG) analysis was performed on STA 449F3 with a mass spectrometry (MS) instrument in order to evaluate the oxidation resistance of the catalysts in a flow of 5% O_2/N_2 (100 mL/min). The desorbed substances (NO/NO_2 (M/e = 30), CO_2 (M/e = 44)) from the evaluated catalyst were monitored by an online MS apparatus.

Temperature-programmed desorption of ethanol (Ethanol-TPD-MS) was carried out using a Microtrac BELCat II with a MS instrument. The carbon catalyst (50 mg) was loaded into a U-shaped fixed-bed quartz micro-reactor and pre-treated for 0.5 h at 120 °C in a pure He flow of 40 mL/min^{-1}. Then, the carbon catalysts were carried out with an ethanol flow rate of 40 mL/min. After that, the temperature was increased from 40 to 600 °C with a ramping rate of 10 °C/min. The desorbed substances (ethanol (M/e = 31)) from the evaluated catalyst were monitored by an online MS apparatus. The temperature-programmed desorption of acetaldehyde (Acetaldehyde-TPD-MS) was similar to Ethanol-TPD-MS. The desorbed substances (acetaldehyde (M/e = 29)) from the evaluated catalyst were monitored by an online MS apparatus.

The surface chemical composition was analyzed with XPS by using an X-ray photoelectron spectrometer (Thermo-Fisher Scientific K-Alpha+), and Al Kα (1486.6 eV) was used as the X-ray source. The binding energy values were obtained with reference to the C1*s* peak from the carbon surface deposit at 284.8 eV.

The NH_3 temperature-programmed desorption (NH_3-TPD-MS) experiment was performed to study the acidity of the catalysts by using a Micromeritics Autochem II 2910 with mass spectrometry (MS). First, 100 mg of catalyst was introduced into the U-shaped tube at 393 K in pure nitrogen flow, then purged with helium for 30 min. After being cooled to 323 K, the sample was pretreated with NH_3 for 1 h to ensure that the acid sites were occupied sufficiently. After that, the samples were heated to 873 K in the pure He flow. The desorbed substances (NH_3 (M/e = 17)) from the evaluated catalyst were monitored by an online MS apparatus.

The Boehm titration method entails the addition of 1 g of carbon material to a solution containing NaOH, Na_2CO_3, and $NaHCO_3$. The mixture was subjected to ultrasonic dispersion for 20 minutes and filtration. The excess alkali in the filtrate was absorbed and titrated with HCl. The determination of acid groups is based on the different neutralized species on the surface of the carrier material: NaOH is assumed to neutralize carboxyl, lactone, and phenolic hydroxyl groups, and Na_2CO_3 for carboxyl and lactone groups, whereas $NaHCO_3$ only for carboxyl groups.

1.5. Calculation methods

Spin-polarized density functional theory (DFT) calculations were performed by using the Vienna ab initio simulation package (VASP) [1-2] and the Perdew–Burke–Ernzerhof (PBE) [3] exchange correlation functional. The projector augmented wave (PAW) method was used to describe the electron–ion interactions. The weak van der Waals interactions between catalyst and adsorbate were handled by the Grimme's DFT-D3 [4] dispersion correction method. A cutoff energy of 400 eV was set for the plane wave basis set. The convergence thresholds for the energy and force were 10^{-5} eV and 0.01 eV/Å, respectively. To avoid the interactions between slabs, a vacuum region of 15 Å was applied along the *z*-direction in the simulated cells. Considering the cell size (cell parameters: 12.16 Å × 12.86 Å × 17.18 Å), we used the 3 × 3 × 1 Monkhorst–Pack *k*-point mesh in reciprocal space.

The adsorption energy was calculated as:

$$\Delta E = E_{\text{total}} - E_{\text{catal}} - E_{\text{int}} \tag{1}$$

where E_{total}, E_{catal}, and E_{int} are the total energies of the catalyst with adsorbed species, pristine catalyst, and isolated adsorbate, respectively. The corresponding Gibbs free energy was calculated as:

$$\Delta G = \Delta E + \Delta ZPE - T\Delta S \tag{2}$$

where ΔZPE and ΔS are the changes in zero-point energy and in entropy, respectively. T is the reaction temperature (543.15 K).

1. Supporting file diagram

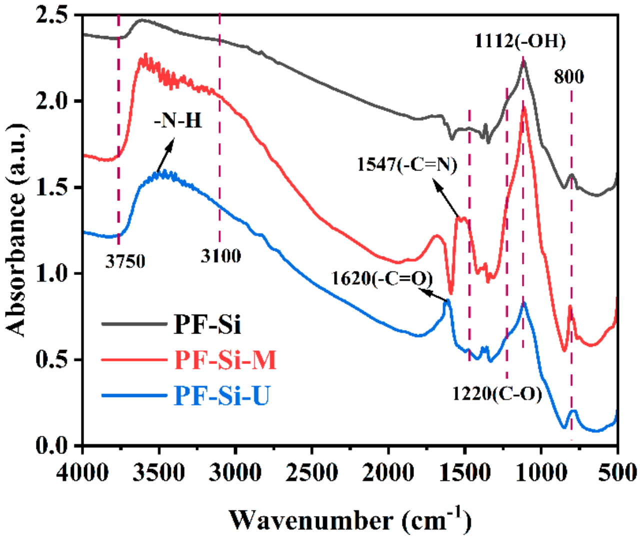

Figure S1. FT-IR spectra of PF-Si, PF-Si-M, and PF-Si-U.

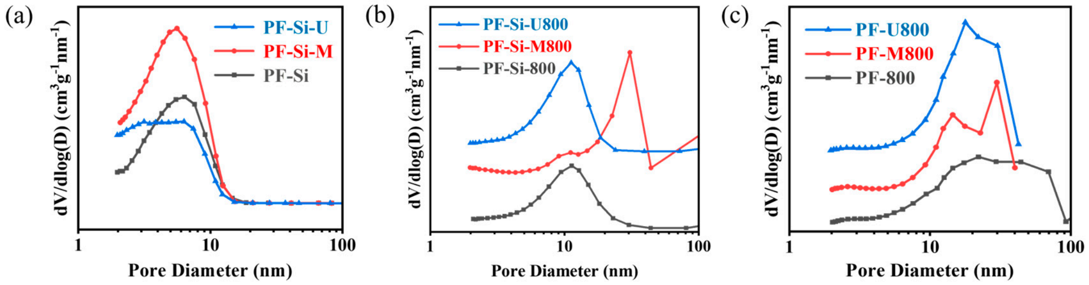

Figure S2. Three stages of the catalyst synthesis process for these samples: Pore size distribution curve.

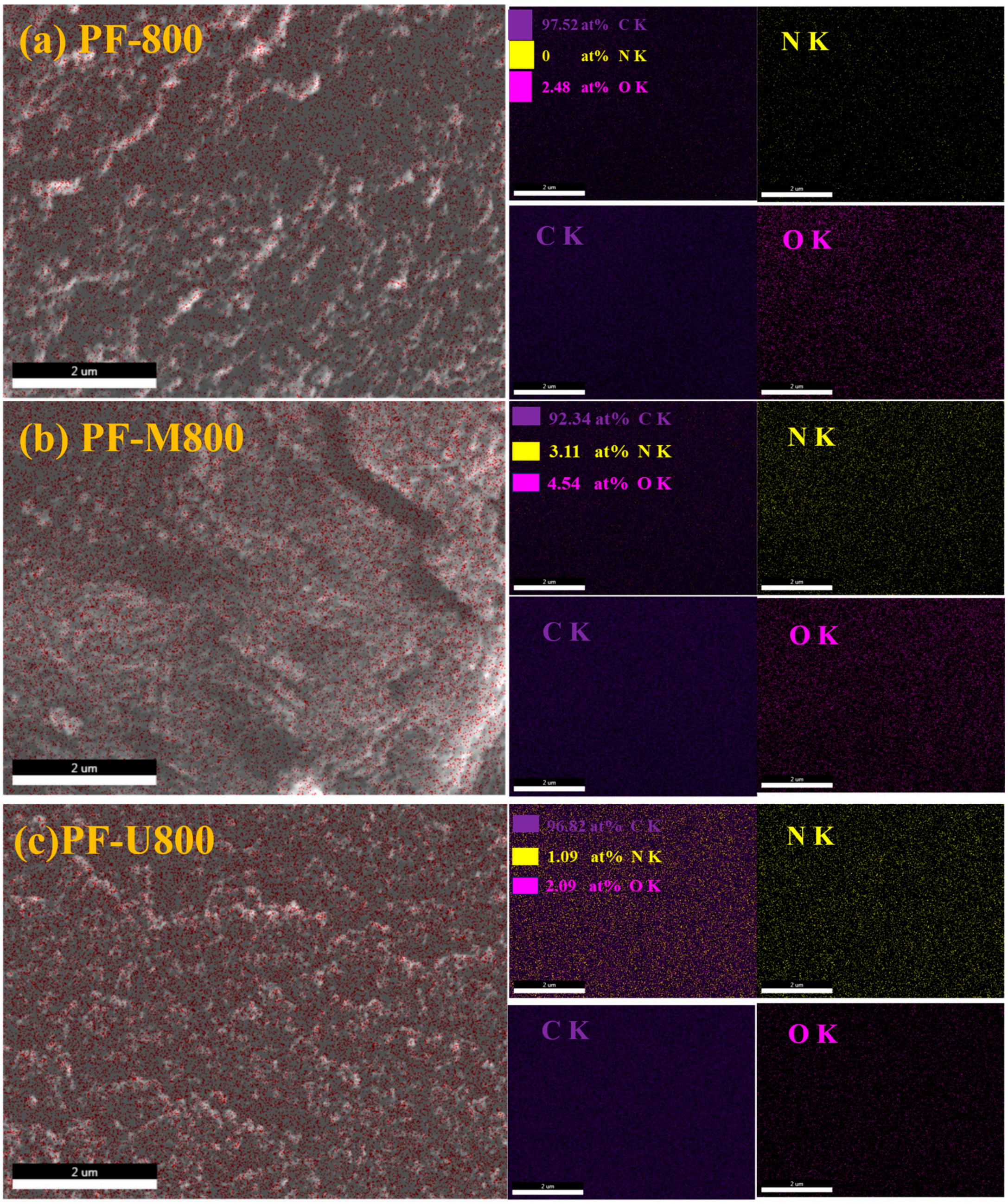

Figure S3. SEM images corresponding to EDX elemental maps of PF-800 (a), PF-M800 (b), and PF-U800 (c).

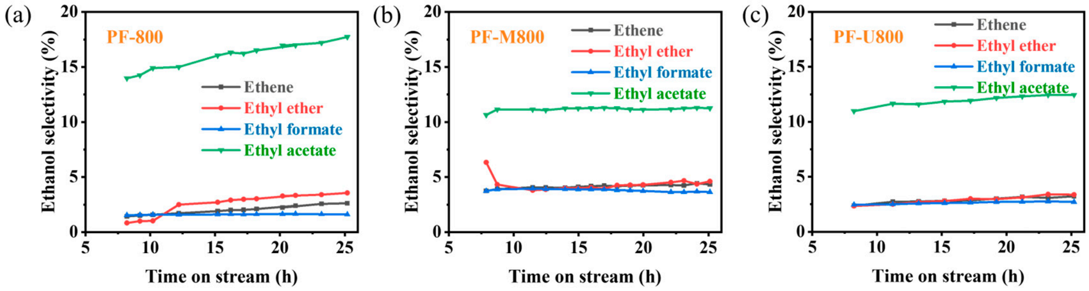

Figure S4. Selectivity of byproducts of PF-800 (a), PF-M800 (b), and PF-U800 (c).

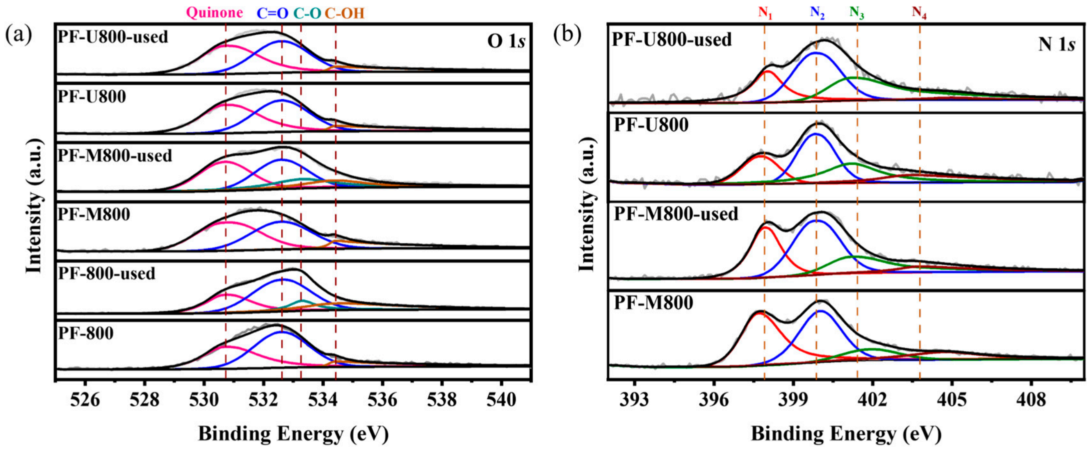

Figure S5. XPS O 1*s* (a) and N 1*s* (b) deconvolution spectra for fresh and used PF-800, PF-M800, and PF-U800.

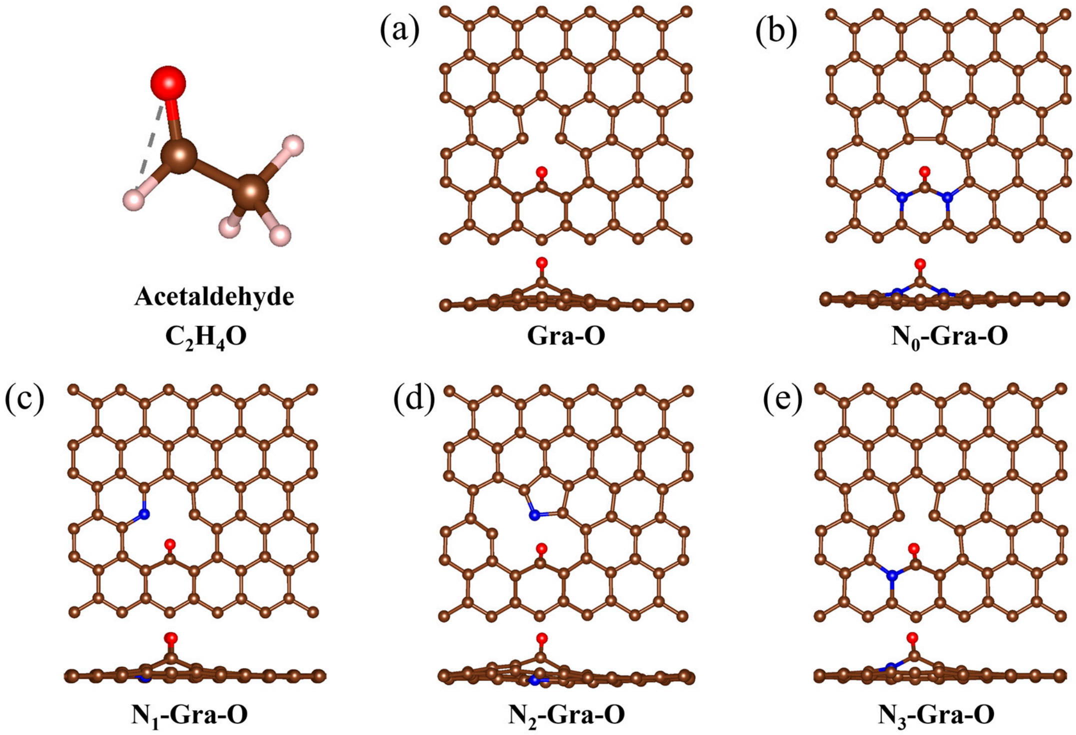

Figure S6 (a)-(e) optimized structures of different catalyst models (top and side views). C: brown; O: red; N: blue; H: white.

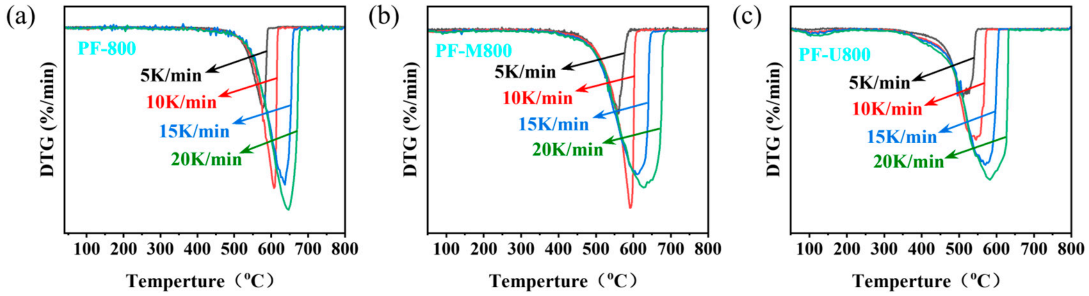

Figure S7. DTG curves at different heating ramps of PF-800 (a), PF-M800 (b), and PF-U800 (c).

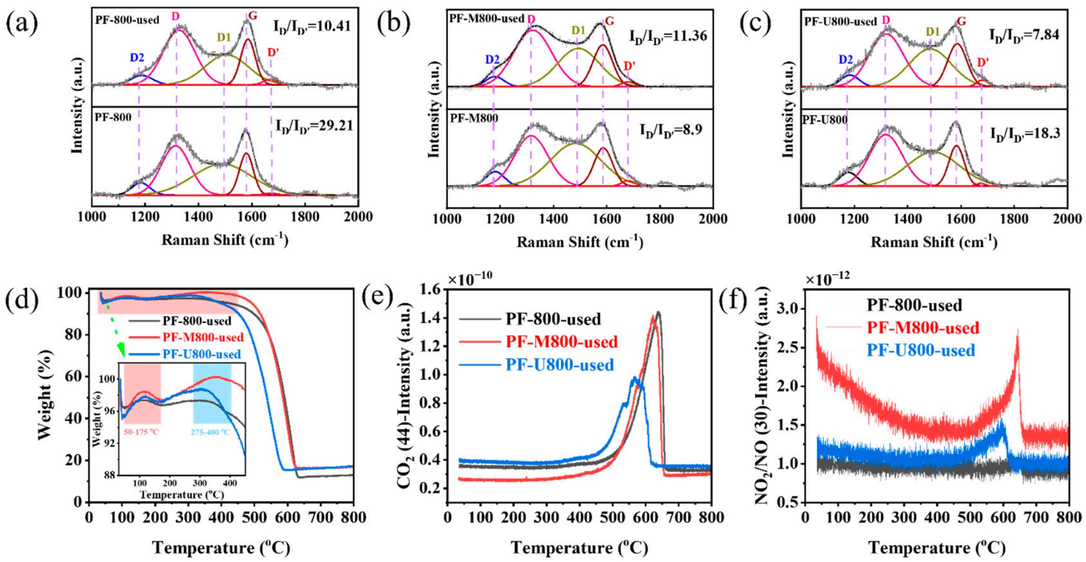

Figure S8. The first-order Raman deconvolution spectra of PF-800 (a), PF-M800 (b), and PF-U800 (c) changed before and after the reaction; TG-MS profiles of the PF-800, PF-M800, and PF-U800 after the reaction (d) and the corresponding MS signals of CO_2 (e), NO and NO_2 (f).

Table S1. Physicochemical properties associated with three stages of catalyst synthesis process.

Sample	S_{BET}(m^2/g)	Vp(cm^3/g)	Dp(nm)
PF-Si	175.1	0.2	4.8
PF-Si -M	324.5	0.4	4.3
PF-Si -U	231.7	0.2	3.7
PF-Si-800	153.9	0.3	7.0
PF-Si-M800	152.9	0.5	12.0
PF-Si-U800	169.2	0.3	7.4
PF-800	974.3	3.4	11.7
PF-M800	941.8	3.6	15.1
PF-U800	999.8	3.6	14.2

Table S2. XPS analysis of content of each element of carbon catalysts before and after the reaction.

Sample	Total C (at.%)	Total N (at.%)	Total O (at.%)
PF-800	94	-	6
PF-800-used	87.1	-	12.9
PF-M800	86.5	8.7	4.8
PF-M800-used	84.9	6.2	8.9
PF-U800	92.81	1.7	5.5
PF-U800-used	88.3	2.3	9.4

Table S3. O 1*s* and N 1*s* XPS analysis results of carbon catalysts.

Sample	Quinone[a]	C=O[a]	C-O[a]	C-OH[a]	Pyridine N[a]	Pyrrole N[a]	Graphitized N[a]	Oxidized N[a]	C=O[b]
PF-800	2.16	3.06	0	0.78	-	-	-	-	5.22
PF-800-used	2.97	6.06	1.21	2.60	-	-	-	-	9.03
PF-M800	2.12	1.93	0	0.74	3.57	3.13	0.87	1.04	4.05
PF-M800-used	3.33	2.93	1.47	1.17	2.17	2.37	1.12	0.50	6.26
PF-U800	2.42	2.35	0	0.73	0.34	0.61	0.48	0.27	4.77
PF-U800-used	4.12	4.04	0	1.24	0.53	0.92	0.78	0.07	8.16

[a] Atomic content from total atomic content × peak area percent (at.%). [b] The sum for the number of quinone and C=O.

Table S4. Desorption amount and desorption temperature of acetaldehyde on various catalysts.

Sample	Peak α/°C	mmol/g	Peak β/°C	mmol/g	mmol/g
PF-800-used	137	0.042	370	0.025	0.067
PF-M800-used	204	0.015	370	0.031	0.046
PF-U800-used	168	0.012	370	0.053	0.065

Table S5. DTG T_{max}, ramp rate, and corresponding activation energy calculated according to Kissinger–Akahira–Sunose equation.

Sample	Ramp rate β (K/min)	T_{max}(°C)	E_a(kJ/mol)
PF-800	5	577.4	110
	10	607.7	
	15	636.7	
	20	646.8	
PF-M800	5	560.7	116
	10	591.2	
	15	611.3	
	20	627.5	
PF-U800	5	511.4	72.6
	10	545.6	
	15	582.7	
	20	599.8	

Table S6. Sample denotations and comparison of the fitting parameters from the peaks fitted in the Raman spectra of carbon-based catalysts before and after the reaction.

Sample	Description	Position(cm^{-1})	FWHM(cm^{-1})	Area
PF-800	D2	1184	80	3089
	D	1314	127	19717
	D1	1489	258	25250
	G	1579	61	8023
	D’	1671	58	311
PF-800-used	D2	1187	94	3106
	D	1327	139	26826
	D1	1505	190	20058
	G	1585	64	10314
	D’	1667	58	1077
PF-M800	D2	1181	80	2951
	D	1316	147	18727
	D1	1483	224	24153
	G	1587	81	7791
	D’	1680	56	798
PF-M800-used	D2	1179	80	3707
	D	1322	168	42711
	D1	1493	186	32092
	G	1585	86	16163
	D’	1680	59	1332
PF-U800	D2	1178	80	3425
	D	1318	145	23141
	D1	1495	230	24285
	G	1583	67	8402
	D’	1673	40	355
PF-U800-used	D2	1183	80	2832
	D	1319	152	23978
	D1	1484	186	21051
	G	1585	84	10888
	D’	1680	50	1022

Table S7. The number of surface functional groups on carbon catalysts.

Sample	-COOH (mmol/g)[a]	Phenolic hydroxyl group (mmol/g)[a]
PF-800	0.265	0.164
PF-800-used	0.442	0.767
PF-M800-used	0.313	0.454
PF-U800-used	0.478	1.072

[a] Measured by Boehm titration method.

References

1. Kresse, G.; Furthmiiller, J. Efficiency of Ab-Initio Total Energy Calculations for Metals and Semiconductors Using a Plane-Wave Basis Set. *Comput. Mater. Sci.* **1996**, *6*, 15.
2. Kresse, G.; Furthmiiller, J. Efficient Iterative Schemes for Ab Initio Total-Energy Calculations Using a Plane-Wave Basis Set. *Phys. Rev. B* **1996**, *54*, 11169.
3. Perdew,J. P.; Burke, K.; Ernzerhof, M. Generalized Gradient Approximation Made Simple. *Phys. Rev. Lett.* **1996**, *77*, 3865.
4. Grimme, S.; Antony, J.; Ehrlich, S.; Krieg, H. A consistent and Accurate Ab Initio Parametrization of Density Functional Dispersion Correction (DFT-D) for the 94 Elements H-Pu. *J. Chem. Phys.* **2010**, *132*, 154104.
